# *Talaromyces marneffei* activates the AIM2-caspase-1/-4-GSDMD axis to induce pyroptosis in hepatocytes

**DOI:** 10.1080/21505594.2022.2080904

**Published:** 2022-05-31

**Authors:** Gang Wang, Wudi Wei, Zhongsheng Jiang, Junjun Jiang, Jing Han, Hong Zhang, Jiaguang Hu, Peng Zhang, Xu Li, Tao Chen, Jinhao He, Zhen Li, Jingzhen Lai, Hao Liang, Chuanyi Ning, Li Ye

**Affiliations:** a Guangxi Key Laboratory of AIDS Prevention and Treatment, School of Public Health, Guangxi Medical University, Nanning, Guangxi, China; b Department of Infectious Diseases, Liuzhou People’s Hospital, Liuzhou, Guangxi, China; c Guangxi Collaborative Innovation Center for Biomedicine, Life Science Institute, Guangxi Medical University, Nanning, Guangxi, China; d Nursing College, Guangxi Medical University, Nanning, Guangxi, China

**Keywords:** *Talaromyces marneffei*, liver damage, pyroptosis, inflammation, opportunistic infection

## Abstract

*Talaromyces marneffei* tends to induce systemic infection in immunocompromised individuals, which is one of the causes of the high mortality. The underlying molecular mechanisms of *T.marneffei*-induced abnormal liver function are still poorly understood. In this study, we found that *T.marneffei*-infected patients could develop abnormal liver function, evidenced by reduced albumin and increased levels of aspartate aminotransferase (AST) and AST/alanine aminotransferase (ALT). *T. marneffei*-infected mice exhibited similar characteristics. *In vitro* investigations showed that *T.marneffei* induced the death of AML-12 cells. Furthermore, we determined that *T.marneffei* infection induced pyroptosis in hepatocytes of C57BL/6J mice and AML-12 cells, demonstrated by the increase of AIM2, caspase-1/-4, Gasdermin D(GSDMD) and pyroptosis-related cytokines in *T.marneffei*-infected mice/cells. Importantly, cell death was markedly suppressed in the presence of VX765 (an inhibitor of caspase-1/-4). Furthermore, in the presence of VX765, *T.marneffei*-induced pyroptosis was blocked. Nevertheless, necroptosis and apoptosis were also detected in infected animal model at 14 days post-infection. In conclusion, *T.marneffei* induces pyroptosis in hepatocytes through activation of the AIM2-caspase-1/-4-GSDMD axis, which may be an important cause of liver damage, and other death pathways including necroptosis and apoptosis may also be involved in the later stage of infection.

## Introduction

*Talaromyces marneffei* (*T. marneffei*) was included in the list of “critical pathogens” released at ameeting of the World Health Organization expert group on anti-fungal diseases [1]. *T.marneffei*, previously known as *Penicillium marneffei*, is the only dimorphic member of the species and is an emerging pathogenic fungus that can cause afatal systemic mycosis known as talaromycosis (formerly called penicilliosis) [2,3]. This disease is endemic in southeast Asia and southern China, and about 50,000 people living with HIV are infected with *T.marneffei* everyyear in high-risk regions [4,5]. Southern provinces accounted for 99% of talaromycosis cases in China, 43% of which were in Guangxi. Among HIV+ populations, the proportion of *T.marneffei* infection is 9–18%, and the mortality rate is 25per 100person-months [95% confidence interval: 21.5–26.7] [4,6,7]. Increasing globalization, rising HIV prevalence, and emerging iatrogenic immunodeficiency conditions mean that the disease burden attributed to talaromycosis will continue to rise.

Talaromycosis is the leading cause of death in patients with advanced HIV disease and is associated with amortality of more than 30% if not given timely and appropriate systemic antifungal therapy [8–11]. Based on the natural route of infection, the most commonly involved organ is the lung, but the liver, although frequently overlooked, is also acommonly involved organ [12–14]. Previous studies and clinical observations have shown that *T.marneffei* infection may cause liver damage. While the liver can show different pathological alterations depending on immune status after *T.marneffei* infection, aspectrum of pathological damage to the liver has been observed in cases of hepatic talaromycosis in some clinicopathological studies [15,16]. However, the specific pathological mechanism is not clear. Currently, the National Free Antiretroviral Treatment Program requires that the start of antiretroviral treatment should be delayed when liver function abnormalities are greater than level 3, but this increases the risk of opportunistic infections as the restoration of the patient’s immune system will be delayed [17]. Therefore, it is clinically imperative to resolve *T.marneffei-*mediated liver damage.

Among the numerous factors linked to inflammation-induced liver injury, pyroptosis is gaining increasing attention. Pyroptosis is an acute inflammatory response triggered by either caspase-1 or caspase-4/-11, caspase-5, and pro-inflammatory cytokine maturation [18–20]. Several pathogens have been demonstrated to induce liver damage through the stimulation of pyroptosis. NLRP3 inflammasome is activated by *Aspergillus flavus* and provoked hepatocyte pyroptosis and oxidative stress, which led to liver damage [21,22]. *Aspergillus fumigatus* (*A.fumigatus*) enhanced the expression of pyroptosis-related proteins in macrophages [23]. *Pseudomonas aeruginosa (P.aeruginosa)* activated the AIM2 receptor through DNA released from host mitochondrial damage, thereby up-regulating the inflammasomes and causing liver damage [24,25]. In addition, the TNF-α signaling pathway and lipid metabolism pathways are also involved in liver injury in some hepatic diseases. Accumulating evidences suggested that innate immune sensors, including NOD-like receptor (NLR) family members (NLRP1B, NLRP3, and NLRC4, etc.), and other non-NLR receptors, such as AIM2, played important roles in the pathophysiology of infectious, inflammatory, and autoimmune diseases. The DNA released during microbial lysis directly binds to AIM2, resulting in oligomerization with ASC and caspase-1 to form afunctional inflammasome complex that promotes caspase-1-dependent pyroptosis by activating the AIM2/caspase-1 pathway [26–29]. In this study, we first analyzed epidemiological data to quantify markers of liver damage in HIV/*T.marneffei* co-infected patients. Afterward, we explored the mechanism of liver injury caused by *T.marneffei* in an *invitro* model using cultured hepatocytes and in an *invivo* model using immunocompetent mice. Our study explains clinical changes in the liver function of *T.marneffei* positive patients, in order to help healthcare providers optimize the clinical management of HIV/*T.marneffei* co-infected patients.

## Materials and methods

### Ethics statement

Guangxi Medical University Research Ethics Committee approval was obtained (approval number: 2019-SB-065), and all patients gave their informed consent. Animal care and protocols were approved by Animal Welfare Committee of Guangxi Medical University (approval number: 202,006,001).

### Fungal strain

The *T.marneffei* strain used in this experiment was isolated from an HIV/*T.marneffei* co-infected patient and identified by PCR amplification and 16S-23S rRNA internal transcribed spacer region (ITS) sequences analysis. The strain was incubated on Sabouraud’s dextrose agar (SDA) and Potato dextrose agar (PDA) medium and maintained at 4℃. *T.marneffei* conidia were cultured on SDA and PDA at 25℃ for 10–14 days and collected by flooding the surface of aculture with phosphate-buffered saline (PBS) and counted with ahemocytometer. Lactophenol cotton blue staining was used to observe *T.marneffei* conidia and fungal morphology.

### Cells culture and treatments

AML-12 (normal mouse hepatic cells) was obtained from the Cell Bank of Shanghai Institutes for Biological Sciences, Chinese Academy of Sciences and cultured in Dulbecco’s Modified Eagle’s Medium/F12 (DMEM/F12) (GIBCO, USA) containing 10% fetal bovine serum, 10 µg/ml insulin, 5.5 µg/ml transferrin, 5 ng/ml selenium and 40 ng/ml dexamethasone. Cells in this medium were cultured at 37℃ in ahumidified atmosphere containing 5% CO_2_ and seeded in 6-well plates at adensity of 2 × 10^5^ cells/well or in 24-well plates at adensity of 5 × 10^4^ cells/well, respectively. For stimulation, hepatic cells were treated with *T.marneffei* [(Multiplicity of Infection, MOI) = 3)] for 12 h, 24 h and 48 h, respectively.

### Animal experiments

Six to eight-week-old male C57BL/6J mice were purchased from Guangxi Medical University Laboratory Animal Center. Mice in the infection group were injected intravenously with 200 microliters of *T.marneffei* conidia at adose of 10^5^ colony-forming units per microliter, and the control group was injected with the same volume of saline. liver tissues and plasma were collected after 3, 7 and 14 days of infection for following experiments.

### Transmission electron microscopy (TEM)

Cells or tissue samples were fixed overnight at 4℃ in 3% glutaraldehyde. The cell pellets or tissues were washed three times with PBS and subsequently stained with osmium solution for 1 h at 4℃. The stained samples were washed three times with PBS, and then dehydrated with 50%, 70%, 90% acetone, respectively for 15 min, finally with 100% acetone three times, 15 min each time. The samples were embedded with Epon-618 resin, with 2 h in amixture of acetone and embedding agent (1 :1), 3 h in amixture of acetone and embedding agent (1 :3), overnight in embedding agent at 37℃, and 60℃ incubation for 48 h. The embedded samples were cut into 60–80 nm sections, and stained with 2% uranium acetate saturated alcohol solution and lead citrate for 15 min respectively. The sections were examined using an H-7650 transmission electron microscope (Hitachi, Japan).

### Immunostaining and histology

Liver tissue sections from each group of mice were prepared and fixed in 4% paraformaldehyde at 4℃ overnight and embedded in paraffin. Paraffin sections (3–4 μm) were stained with hematoxylin–eosin (H&E) reagent, periodic acid-Schiff (PAS), and Gomori’s methenamine silver (GMS) separately. After mounting, the sections were observed by an inverted light microscope (Nikon Diaphot Microscope, Japan).

### Total RNA extraction, library construction and sequencing

The C57BL/6J mice infected with *T.marneffei* at 3, 7 and 14 days were compared with control group. Total RNA was extracted from mouse liver tissue using Trizol reagent (Invitrogen, USA) according to the manufacture’s protocol. RNA quality was assessed on an Agilent 2100 Bioanalyzer (Agilent Technologies, USA). Then, mRNA was enriched by Oligo(dT) beads, while prokaryotic mRNA was enriched by removing rRNA by Ribo-ZeroTM magnetic kit (Epicenter, USA). Subsequently, the enriched mRNA was fragmented into short fragments using fragmentation buffer and reverse transcripted into cDNA, which was purified with QiaQuick PCR extraction kit (Qiagen, Netherlands), and ligated to Illumina sequencing adapters. The library was sequenced using Illumina Novaseq6000 System (Guangzhou, China). In this study, atotal of 21 samples at 3 time points were sequenced, with 3 control samples and 4 *T.marneffei*-infected samples at each time point.

### Differential analysis of transcriptome

The reads were obtained from the sequencing machines and were further filtered by fastp software (version 0.18.0) to get high quality clean reads. Paired-end clean reads were mapped to the reference genome using HISAT2. 2.4 to calculate read counts for each unigene. Differentially expressed genes (DEGs) between different experimental conditions were identified using atwo-sided Wald test in DESeq2 package, with statistical significance set as |log_2_ fold of change| > 1 and adjusted *P*-value <0.05. Because the DESeq2 package requires input in the form of raw count, which is normalized by the estimateSizeFactors function, we did not calculate reads per kilobase of exon model permillion mapped reads before this. The heatmap was constructed using the pheatmap Rpackage to inflect the expression intensity and direction of the DEGs in different time points. The volcano plots were constructed based on ggplot2 R package to exhibit the DEGs whose |log_2_ fold of change| were larger than 1.

### Function enrichment analysis

The potential function of DEGs among different times infected with *T.marneffei*, modules enrichment analyses were predicted using Clusterprofiler Rpackage. Genes with |log_2_ fold of change| > 1 and adjusted *P*-value <0.05 were selected for Kyoto Encyclopedia of Genes and Genomes (KEGG) and Gene Ontology (GO) analysis. Gene set enrichment analysis (GSEA) was performed on RNA expression profiling using the Rpackage ‘clusterProfiler’.

### Flow cytometry analysis

Flow cytometry was used to detect cell damage. Briefly, after stimulationwith *T.marneffei* for 24 h, 48 h and 72 h, the samples were harvested to stain with propidium iodide (PI, BD Biosciences, USA), which was used at afinal concentration of 500 ng/ml. Then samples were run and analyzed using Cyto FLEX2 (Beckman Coulter, USA).

### Assessment of lactate dehydrogenase (LDH)

Released LDH in culture supernatants from damaged cells was measured with LDH assay kit (Nanjing Jiancheng Bioengineering Institute, China), and the LDH activity in cell supernatants was calculated according to the instructions. The optical density (OD) value was read at 440 nm with amicroplate reader (BioTek, USA).

### Analysis of alanine aminotransferase (ALT) and aspartate aminotransferase (AST)

ALT assay kits and AST assay kits (Nanjing Jiancheng Bioengineering Institute, China) were purchased to examine ALT and AST released by plasma and hepatic cells supernatants.

### Primers designing and reverse transcription quantitative PCR (RT-qPCR) analysis

Primers for the amplification of *Aim2*, c*aspase-1*, *caspase-4*, *Il-1β*, *Il-18*, *Tnf-α*, *Gsdmd*, *Gapdh and β-actin* were designed in PrimerBank website (https://pga.mgh.harvard.edu/primerbank/). Primers were optimized prior to quantification experiments using RT-qPCR. Primer sequences were shown in Table S1. Total RNA was purified using Trizol reagent and chloroform extraction, followed by precipitation in isopropyl alcohol and 75% ethanol. Reverse transcription was performed using areverse transcription kit (TAKARA, Japan). RT-qPCR was performed using SYBR Premix ExTaq^TM^(TAKARA, Japan) by StepOnePlus Real-time PCR system (Thermo Fisher Scientific, USA).

### Protein extraction and western blot analysis

AML-12 cells and liver tissues were harvested and subjected to protein extraction using cell lysis buffer (CST, USA) according to the manufacture’s protocol. After protein extraction, protein concentrations were determined using BCA protein concentration assay kit (Beyotime, China). An equal amount of protein extracted from AML-12 cells or liver tissues were separated by sodium dodecyl sulfate-polyacrylamide gel electrophoresis (SDS-PAGE), and transferred to polyvinylidene difluoride membrane. After blocking with 5% nonfat milk, membranes were incubated at 4°C overnight with the specific primary antibodies, such as caspase-1, cleaved caspase-1, gasdermin D(GSDMD), cleaved-GSDMD and β-actin, and then incubated for 1 h with the corresponding secondary antibody. Images were developed using the Odyssey CLX two-color infrared laser imaging system (LI-COR, USA).

### Lentivirus transfection

The lentivirus vector with *caspase-1* siRNA (ID NM_009807) was constructed by Genechem (Shanghai, China). *caspase-1* gene expression was interfered using *caspase-1*-RNAi-lentivirus transfection technology. The sequences were designed and synthesized as follows: RNAi-*caspase-1*-F:5’-CCGGGGGCAAAGAGGAAGCAATTTACTCGAGTAAATTGCTTCCTCTTTGCCCTTTTTG-3’, RNAi-*caspase-1*-R:5’-AATTCAAAAAGGGCAAAGAGGAAGCAATTTACTCGAGTAAATTGCTTCCTCTTTGCCC-3’. Non-transfected hepatic cells were used as the blank control, and negative control (NC) was transfected with empty lentivirus vector. The *caspase-1*-RNAi-lentiviruses were transfected for the knock down (KD) groups.

### Data analysis and statistical analysis

For RT–qPCR data, relative gene expression was defined as aratio of target gene expression versus *Gapdh* and *β-actin* genes expression. The relative levels of RNA were calculated using the comparative CT (2 ^−ΔΔ Ct^) method.

For western blot data, relative protein levels were calculated as the density ratios of target protein to β-actin, that is, the gray density of the target protein band was divided by that of the internal reference protein of the corresponding sample (target protein/β-actin) to make the corresponding statistical data. Protein quantification was performed using Image Jsoftware.

For statistical analysis, quantitative data with normal distribution were presented as mean ± standard deviations (SD), and nonnormal data were presented as median and the interquartile range. Comparisons were performed by Student’s *t*test or one-way ANOVA analysis for normally distributed data, and Wilcoxon rank-sum test for nonnormal data. The results of *P*-value of <0.05 was considered statistically significant, *P* < 0.01 indicated astatistically very significant difference, while *P* < 0.001 indicated astatistically extremely significant difference. SPSS 23, Rstudio and GraphPad Prism 8 was used to analyze. Furthermore, propensity score matching (PSM) analysis was used to reduce selection bias and potential confounding effects caused by CD4+ Tlymphocytes, leukocytes, and platelets, etc. The HIV-1 mono-infected group and HIV/*T.marneffei* co-infected group were matched in a1 : 1 ratio using the nearest neighbor matching technique without replacement, with acaliper of 0.06.

## Results

### T.marneffei *infection resulted in abnormal liver function in HIV/T*. marneffei *co-infected patients*

In order to investigate the existence of abnormal liver function in *T.marneffei*-infected patients, we collected samples from 200 HIV-positive patients from Liuzhou People’s Hospital for analysis. Laboratory tests were launched before initiating antifungal therapy to examine the differences in biochemical indicators between the two groups. The PSM was performed to compensate for selection bias. In total, all routine blood variables were similar between the contrast and noncontrast groups via PSM. When the blood routine indexes were comparable between the two groups, serum albumin decreased by 5 g/L in HIV/*T.marneffei* co-infected patients. In patients with co-infections, AST increased to 59 U/L (interquartile range: 38, 142), exceeding the upper normal limit, and AST/ALT increased from 1.52 to 2.63 (Table S2 and Figure S1). These data indicate impaired liver function in patients with co-infections. Therefore, the results of the epidemiological study showed that *T.marneffei* infection might contribute to abnormal liver function.

### T.marneffei *infection resulted in liver damage in C57BL/6J mice*

**Figure 1.**
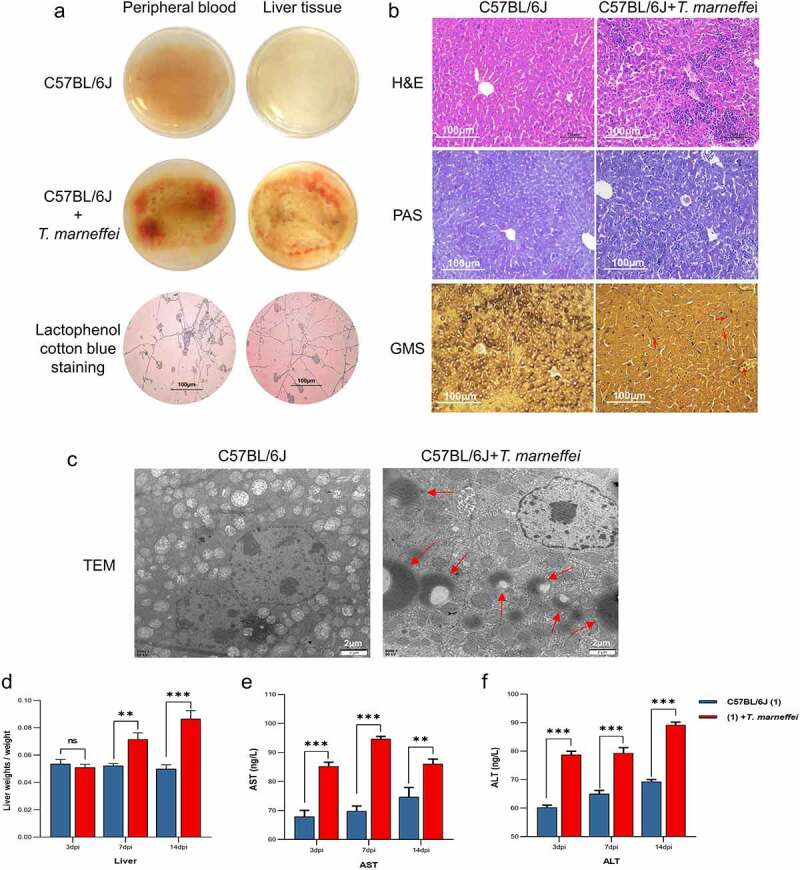
*T.marneffei* infection resulted in liver injury in C57BL/6J mice. (a) Whole blood and liver tissue homogenates were incubated at 28℃ for 3d on PDA plates. Large numbers of hyphae and conidia were found on the surface of the medium in the *T.marneffei* infection group. (b) H&E staining, PAS staining and GMS staining, 200 × . The infection group exhibited obvious inflammatory lesions, inflammatory cell infiltration, disordered arrangement of cell cords, unclear structure and hepatocyte necrosis. In GMS staining, the red arrows point to *T.marneffei*. (c) TEM, the red arrows pointed to *T.marneffei*. (d) Liver index (liver weight/body weight) between control group and infection group at 3, 7 and 14 dpi, respectively. (E and F) AST and ALT were determined to estimate hepatic damage. (*P<0.05, **P<0.01, ***P<0.001).

To confirm the epidemiological findings, we constructed a*T.marneffei*-infected model using 6–8 week old male C57BL/6J mice. At 3, 7 and 14 days post-infection (dpi), whole blood, plasma and liver tissues were collected for analysis. Whole blood and liver tissue homogenates were incubated on PDA plates, and alarge number of conidia were found in the *T.marneffei*-infected group(Figure1a). H&E staining of the control group liver tissue showed aregular tissue architecture, alack of fat accumulation in hepatocytes, and the absence of inflammatory cells infiltration. In contrast, the infection group showed inflammatory lesions, mainly manifested as inflammatory cell infiltration (such as monocytes/macrophages, mDC cells, and CD4+ Tcells, etc.), the disordered arrangement of cell cords, and an unclear structure. Using PAS and GMS staining, round to oval sporangia with internal divisions consistent with *T.marneffei* were visible both free and within liver tissue (Figure1b). TEM revealed intracellular disturbances: distension of the endoplasmic reticulum; mitochondrial enlargement, swelling and vacuolar degeneration of the mitochondrial cristae; and alighter matrix and fewer cristae. *T.marneffei* were clearly present in liver tissue (Figure1c). The liver index (liver weight/body weight) indicated that the liver of the infection group increased significantly by 1.73-fold at 14 dpi (Figure1d). Plasma levels of AST and ALT were significantly increased (more than 1-fold) in the infection group at 3, 7 and 14 dpi, respectively (Figure1e-f).

### Transcriptomics profiles and the expression levels of cell death-related pathways in *T. marneffei*-infected liver tissues

**Figure 2.**
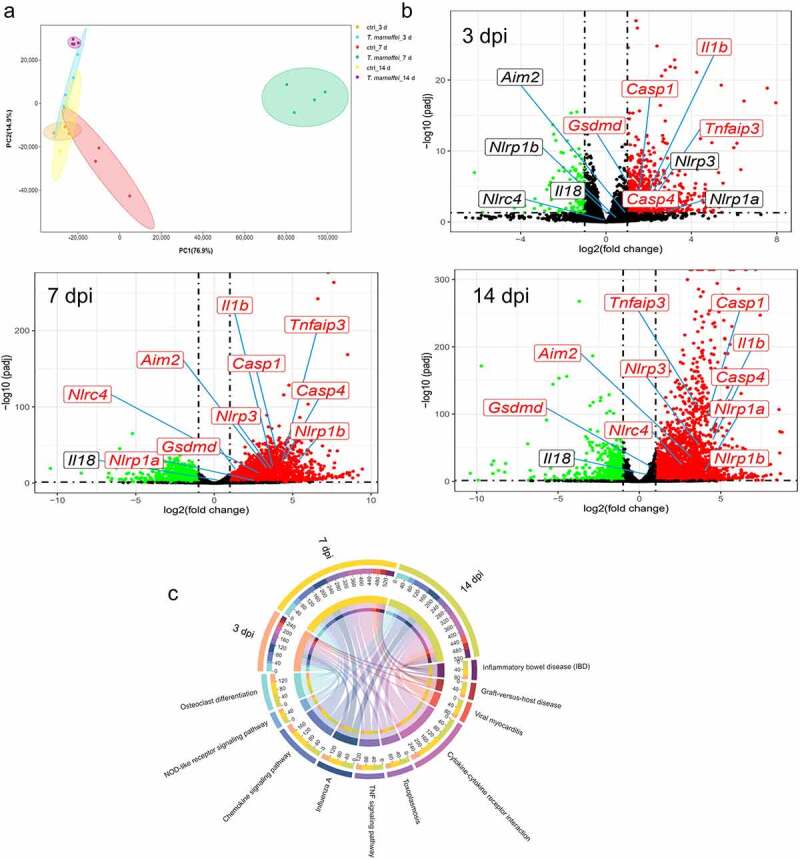
RNA-Seq analysis of C57BL/6J mice liver tissues at 3, 7 and 14 days post *T.marneffei* infection. (a) Principal component analysis on RNA-seq data. The data showed clear grouping of replicate samples for same developmental stage and infection groups. PC1 on the X-axis captures the greatest variation and accounts for 77% of the total variance around the PCs, while PC2 on the Y-axis captures thesecond greatest variation and accounts for 15% of the total variance around the PCs. (b) Volcano plots of DEGs at 3, 7 and 14 dpi. The mean expression level for control group vs. infection group was assessed for fold-change (X-axis) vs *P*-value (Y-axis) by *Wald* test based on DEseq2 package. Each gene is represented by afilled circle. Red indicates ahigher expression level in infected group compared with control group, while green means the opposite. The genes marked in the figures are: *Nlrp1a, Nlrp1b, Nlrp3, Nlrc4, caspase-1, caspase-4, Aim2, Il-1β, Il-18, Tnf-α, Gsdmd*. (c) the chord diagram. The abundance of top 10 KEGG pathways according to adjusted *P*-value in the different time points was exhibited.

We performed RNA-seq analysis of liver tissue to explore the molecular profile of C57BL/6J livers after *T.marneffei* infection. Mice were divided into the infection and control groups by injection of *T.marneffei* conidia and normal saline. The Principal Component Analysis (PCA) was used to describe the differences between different groups of samples (Figure2a). Atotal of 931 (at 3 dpi), 4749 (at 7 dpi) and 3816 (at 14 dpi) differentially expressed genes were obtained based on the threshold chosen (|log_2_ Fold of change| > 1 and adjusted *P*-value <0.05) (Figure2b). Then, the underlying functions of the DEGs in each time point were predicted via Clusterprofiler Rpackage. The DEGs were significantly involved in immune system process, regulation of immune system process (biological process, BP), cytoplasm, cytoplasmic part, intracellular part (cellular component, CC), protein binding and enzyme binding (molecular function, MF) (Figure S2). Figure S3 showed the KEGG enrichment analysis in metabolism, genetic information procession, cellular processes, etc. Figure S4 displayed the top 10 GO and 15 KEGG pathways according to adjusted *P-*value, and the most enriched KEGG included pathways in infectious diseases, immune system, and signal transduction. The chord diagram showed the abundance of top 10 KEGG pathways according to adjusted *P*-value at 3, 7 and 14 dpi (Figure2c). These DEGs, regulated by *T.marneffei* infection, were tightly associated with several signaling pathways, including the NOD-like receptor signaling pathway, TNF signaling pathway, and cytokines-cytokines receptor interaction, etc., indicating that *T.marneffei* infection significantly promoted the inflammatory response of liver tissue.

**Figure 3.**
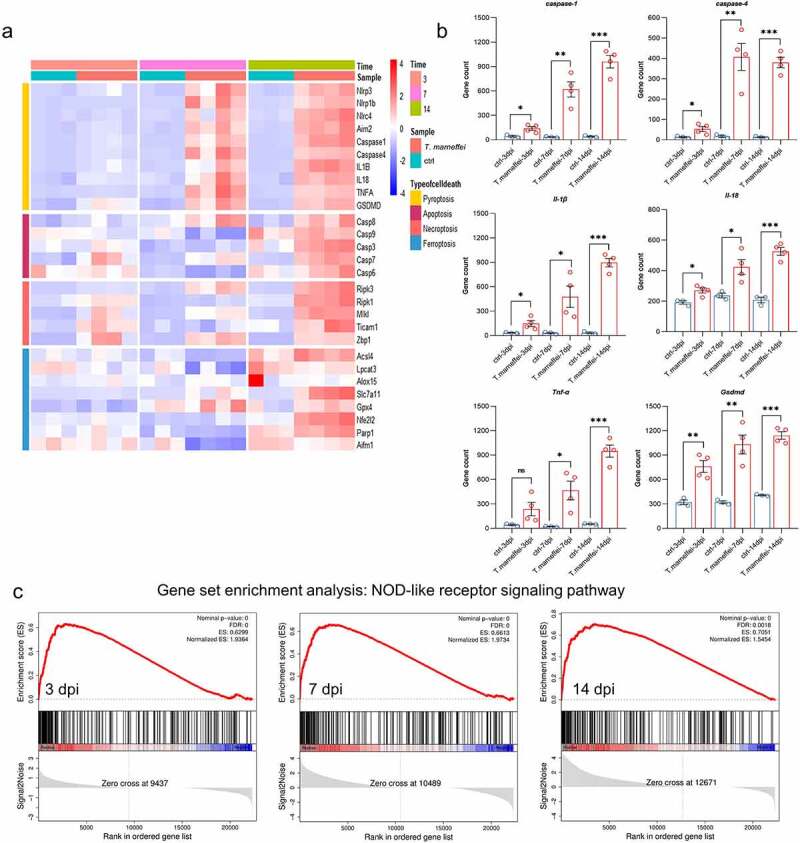
The expression levels of pyroptosis associated factors. (a) Heatmap of genes involved in different types of cell death. Red represents high expression, and blue represents low expression. (b) the mRNA expression level of different pyroptosis related genes. All of these genes are involved in the NOD-like receptor signaling pathway. (c) Results of GSEA analysis. Pyroptosis related genes were all enriched in the NOD-like receptor signaling pathway in different time points when C57BL/6J mice infected with *T.marneffei*. Enrichment score (ES) and FDR were shown. Red curve indicates enrichment score (ES), the gray shadow at the bottom represents the Log_2_ fold of change value of each gene. (*P< 0.05, **P< 0.01, ***P< 0.001).

Based on the KEGG pathway analysis, we further analyzed the pathways related to infection, inflammation and signal transduction, as well as cell death-related pathways that may lead to liver damage. After RNA-seq read count data were obtained, the differences in transcription factors were calculated using the DEseq2 package, which was fitted with amodel for anegative binomial distribution, and hypothesis testing was performed using the *Wald* test. As shown in Figure3a, the transcriptional levels of pyroptosis-related factors were significantly increased, such as *caspase-1* from 2.24-fold at 3 dpi to 22.80-fold at 14 dpi, and *Gsdmd* from 1.38-fold to 2.21-fold, respectively. The activation time of the apoptosis pathway was later, and there was no difference in the expression in the early stage, *caspase-3* was only up-regulated by 0.61-fold at 14 dpi, indicating that apoptosis may just function at later times of infection (Figure S5). The expression of key factors of ferroptosis showed the opposite tendency, that is, the *Gpx4* was significantly up-regulated (27% at 14 dpi) when C57BL/6J mice were infected with *T.marneffei*, indicating that the ferroptosis might not occur (Figure S6). The expression levels of necroptosis associated factors (*Ripk1/3, Mlkl, Ticam1, Zbp1*) were also upregulated, but the change level was lower than the up-regulated level of pyroptosis-related factors (Figure S7). Therefore, we deduced that pyroptosis may be the dominant form of cell death in the livers of C57BL/6J mice infected with *T.marneffei* but did not rule out asynergistic role played by necroptosis. The bar plots showed the expression levels of pyroptosis associated factors (Figure3b), and the different inflammasomes (*Nlrp1b, Nlrp3, Nlrc4* and *Aim2*), upstream stimulatory factors of pyroptosis, were shown in Figure S8. We then employed the GSEA approach, aconventional method for identifying pathways associated with gene expression (Figure3c). The enrichment score of the NOD-like receptor signaling pathway was higher than 0.6 at three time points, and the core genes in this gene set were highly expressed in the infected group. However, the ES of the apoptosis pathway (mmu04210) was relatively low (0.390), and the difference was not statistically significant (*P.adjust* = 0.052) between the infection and non-infection groups. Similar to apoptosis pathway, necroptosis pathway (mmu04217) showed alow ES value (0.386), and the difference was not statistically significant (*P.adjust* = 0.066) between the two groups. In addition, GSEA did not find enriched pathways related to ferroptosis and autophagy.

### T.marneffei *up-regulated the expression levels of pyroptosis-related genes in C57BL/6J mice*

**Figure 4.**
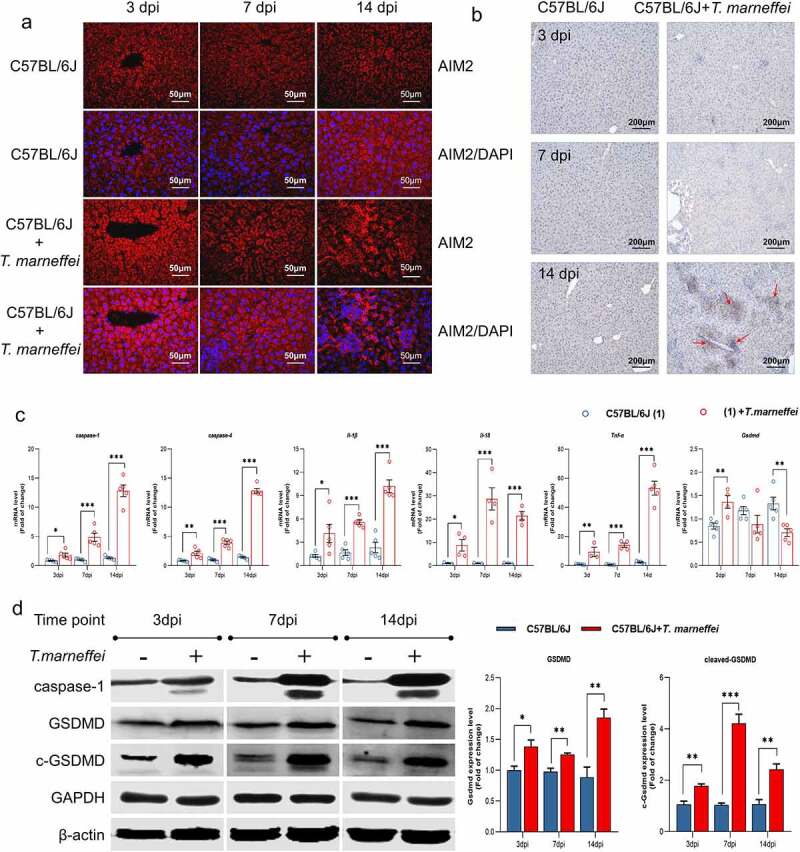
The expression level of pyroptosis associated factors in liver tissue of *T.marneffei*-infected C57BL/6J mice. (a) Immunofluorescence result of the infected group vs. control group in different time points. (b) Representative immunohistochemistry images showing increased staining of caspase-1 protein expression at 14 din samples from *T.marneffei*-infected mice. The red arrows point to increased caspase-1 protein. (c) the transcriptional level of different pyroptosis related genes. *caspase-1/-4*, *Il-18* and *Tnf-α* were up-regulated at 3 dpi, and continued to rise with ongoing infection, while *Gsdmd* exhibited higher level at 3 dpi, and the expression significantly decreased at 14 dpi. (d) the protein expression level of pyroptosis critical proteins by western blot. (*P< 0.05, **P< 0.01, ***P< 0.001).

Afterward, we verified that the pyroptosis related factors were induced in the liver of *T.marneffei*-infected mice. Immunofluorescence imaging revealed the increased expression of AIM2 in *T.marneffei*-infected liver tissue at 3, 7 and 14 dpi (Figure4a). To determine the critical factor of pyroptosis, we used immunohistochemistry to detect caspase-1 in mice livers. As shown in Figure4b, there was increased caspase-1 within the inflammatory infiltrate foci and parenchymal cells in the liver of *T.marneffei-*infected C57BL/6J mice at 14 dpi. RT-qPCR showed that the mRNA levels of *caspase-1/-4*, *Il-18* and *Tnf-α* were upregulated at 3 dpi, and continued to increase with ongoing infection. *Gsdmd* showed asignificantly increased level at 3 dpi, but was significantly decreased at 14 dpi (Figure4c). The protein expression profile of caspase-1 was consistent with the mRNA profile, while the level of GSDMD and cleaved-GSDMD gradually increased from 3 dpi to 14 dpi (Figure4d). These results were consistent with the trends identified by RNA-seq analysis, suggesting that proteins central to pyroptosis may play apathological role during acute *T.marneffei* infection.

### *T.marneffei* up-regulated the expression levels of pyroptosis-related genes in AML-12 cells

**Figure 5.**
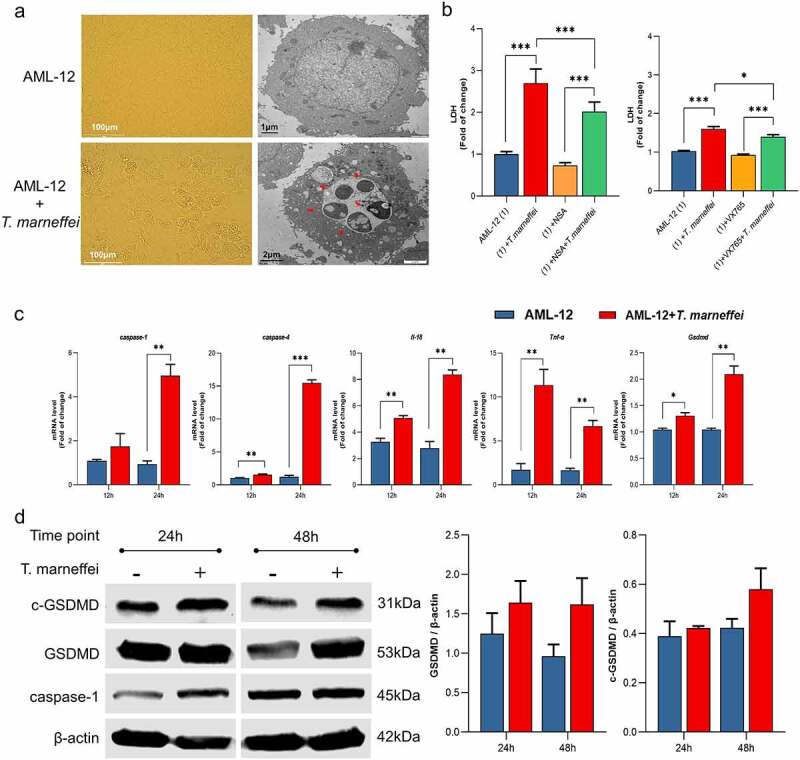
The expression levels of pyroptosis associated factors in AML-12 cells. (a) Light and transmission electron micrographs of AML-12 cells infected by *T.marneffei*. There was asignificant increase in cell death after 48 hof *T.marneffei* infection, and *T.marneffei* conidia were found inside the AML-12 cells. The red arrows pointed to *T.marneffei*. (b) the cell supernatant was collected to detect the LDH expression after 48 hof infection. The level of cell damage was decreased when treated with VX765 (the inhibitor of caspase-1/-4) and NSA (the inhibitor of GSDMD and MLKL) (c) the mRNA expression level of different pyroptosis related genes. (d) the protein expression level of pyroptosis critical proteins by western blot. (*P< 0.05, **P< 0.01, ***P< 0.001).

We established an *invitro* infection model using the AML-12 cell line to verify the effect of *T.marneffei* on the pyroptosis-related pathway. Light microscopy indicated that there was asignificant increase in cell death after 48 h of *T.marneffei* infection (Figure5a). TEM analysis revealed that *T.marneffei* were found inside the AML-12 cells. In addition, alarge number of cavities were produced; the cells swelled and ruptured, and the cellular contents were released (Figure5a). Figure5b showed that LDH levels were significantly reduced by 24% in culture supernatants when the *T.marneffei* infected cells were pretreated with VX765 (the inhibitor of pyroptosis), indicating that pyroptosis is involved in the cell damage induced by *T.marneffei*. This trend was almost similar to necrosulfonamide (NSA, the inhibitor of GSDMD and MLKL) intervention. Flow cytometry was used to detect the damage of *T.marneffei*-infected AML-12 cells, cell damage was increased significantly after infection with *T.marneffei* (13% at 24 h, 22% at 48 h and 33% at 72 h) (Figure S9a). Then, total RNA and protein were extracted to determine the expression levels of pyroptosis related genes, and the results showed that the mRNA and protein expression levels of key pyroptosis factors (caspase-1/-4, IL-18, TNF-α, GSDMD) in the infected group were higher than in the control group (Figure5c-d). It is worth mentioning that GSDMD protein can induce pyroptosis only after being cleaved and split into cleaved-GSDMD protein by caspase, which may lead to the upregulation of cleaved-GSDMD protein later than the *Gsdmd* gene level. In this study, cleaved-GSDMD protein showed an upward trend only after 48 h of infection. The detection of inflammatory factors upstream of pyroptosis showed that only *Aim2* expression was up-regulated by 26% and 96% after 24 hours and 48 hours of infection, respectively, and the difference was statistically significant(Figure S10).

### Inhibition of pyroptosis decreased the inflammatory response of AML-12 cells

**Figure 6.**
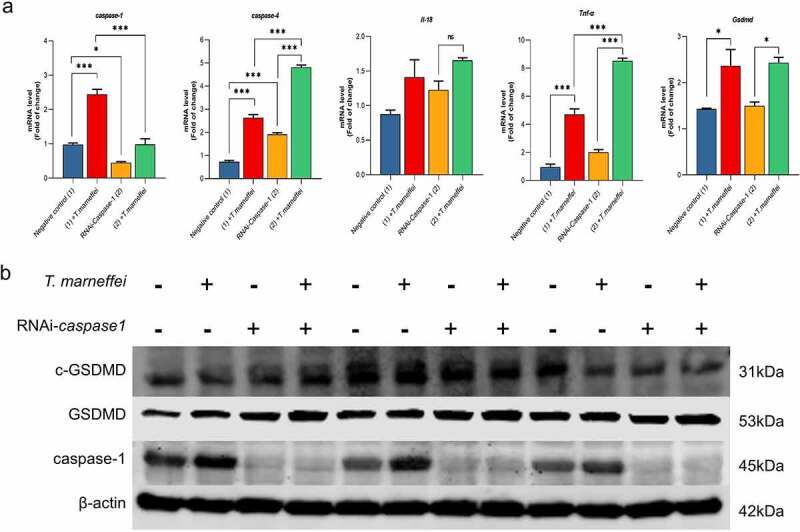
The expression level of pyroptosis associated factors after inhibiting *caspase-1* in AML-12 cells. (a) After lentiviral transfection, the expression of pyroptosis related factors in *T.marneffei-infected* cells was detected at 48 hpost infection. Compared with the cells transfected with NC group, the expression level of *caspase-1* was decreased in RNAi-*caspase-1* group, but the mRNA level of downstream inflammatory factors was not suppressed, and *caspase-4* reflected ahigher expression level. (b) the expression levels of pyroptosis related proteins in *caspase*^*-/-*^ AML-12 cells at 48 hpost infection. Although the expression of *caspase-1* protein was significantly decreased after siRNA transfection, the expression of downstream GSDMD and cleaved-GSDMD proteins were still increased. (*P< 0.05, **P< 0.01, ***P< 0.001).

**Figure 7.**
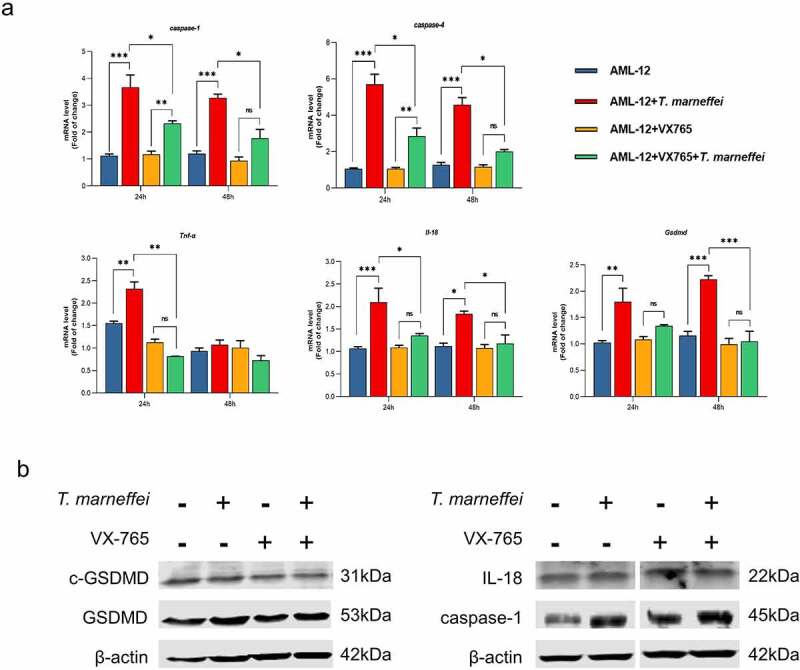
The expression level of pyroptosis associated factors after VX765 pretreatment in AML-12 cells. (a) After VX765 pretreatment, the expression of pyroptosis related factors in *T.marneffei*-infected cells was detected after 48 hof infection. *Caspase-1* and *-4* were all suppressed, and downstream inflammatory factors were also decreased. (b) the expression levels of pyroptosis related proteins in VX765-pretreated AML-12 cells at 48 hpost infection. Compared with infection group, VX765 pretreatment group decreased the expression of caspase-1 protein, and the GSDMD and cleaved-GSDMD proteins also decreased. (*P< 0.05, **P< 0.01, ***P< 0.001).

The role of caspase-1-dependent pyroptosis in *T.marneffei*-induced hepatocytic inflammation was investigated. GFP is afluorescent protein encoded in the lentiviral vector, which is used to determine the transfection efficiency of lentiviral vector. GFP fluorescence in AML-12 cells was observed at 72 h following transfection, which suggested the successful transfection of RNAi-*caspase-1* lentivirus (Figure S11). Compared with the cells transfected with empty lentivirus vectors (NC group), the expression level of *caspase-1* was reduced by 93% in RNAi-*caspase-1* group (Figure S11). However, the mRNA level of downstream inflammatory factors was not suppressed, and *caspase-4* showed ahigher mRNA expression level (0.83-fold increase) (Figure6a), possibly as acompensatory or redundant mechanism. The expression levels of GSDMD and cleaved-GSDMD protein were also elevated, although caspase-1 protein was inhibited (Figure6b). Therefore, we pre-treated cells with VX765, an inhibitor of both caspase-1/-4. The cell viability result demonstrated that 50 μM was the optimal concentration of VX765 (Figure S9b). Therefore, 50 μM VX765 was selected as the concentration used for subsequent experiments. As shown in Figure5b, the LDH cytotoxicity assay showed that LDH levels were significantly reduced by 24% in culture supernatants when the *T.marneffei* infected cells were pre-treated with VX765, indicating that pyroptosis is involved in the cell damage induced by *T.marneffei*. The mRNA expression levels of *caspase-1* and *-4* were all suppressed, and the downstream inflammatory factors *Il-18*, *Tnf-α* and *Gsdmd* were reduced by 35%, 65% and 53%, respectively. (Figure7a). The expression of proteins was consistent with the mRNA results (Figure7b), indicating that when the pyroptosis is inhibited, the inflammatory injury of liver cells will be alleviated.

## Discussion

T.*marneffei* is adimorphic fungus which can infect both HIV/AIDS and non-HIV hosts. At present, the pathogenic mechanism of *T.marneffei* is not well understood. In this study, we found that the infection rate of *T.marneffei* was 10% in HIV-1 infected patients with CD4+ Tcell counts less than 1500 cells/µl, 17% in those less than 200 cells/µl, and 22% in those less than 100 cells/µl. Overall, 25% of HIV/*T.marneffei* co-infected patients had hepatosplenomegaly on admission, while 75% showed abnormal AST levels. Then, we established *invivo* and *invitro* infection models to explore the molecular mechanism of liver damage. The results showed that *T.marneffei* could rapidly invade into the liver and hepatocytes. However, it is still unclear how *T.marneffei* gains entry into hepatocytes. The main function of the liver is to participate in biological metabolism and transformation, and hepatocytes can not only synthesize bile acid, serum albumin, digestive enzymes and various clotting factors, but also participate in the metabolism of lipids, sugars and hormones [30]. In addition, hepatocytes can transform endogenous or exogenous toxins into nontoxic or low-toxic components [31]. At present, the research into pathogen-mediated hepatocyte injury is mainly focused on viral infection [32–34]. Firstly, the innate immunity of hepatocytes provides the first line of defense against pathogens, including hepatitis viruses, and activates both protective direct antiviral and inflammatory responses. For instance, liver damage caused by HBV is due to chronic immune responses by liver-infiltrating immune cells that target infected hepatocytes [35,36]. In the case of fungal infection, many *Aspergillus* species produce Sterigmatocystin (STC), which is arisk factor of liver cancer [37]. It has been reported that tissue examination in mice showed liver cell injury and necrosis after being fed adiet containing STC. Also, STC is considered to be involved in chronic liver disease of people living in Africa [38]. However, few studies indicated that fungi could actively enter liver parenchymal cells. Interestingly, in this study, fungal spores could be cultured on PDA medium in mouse liver tissue infected with *T.marneffei*; TEM revealed disordered liver tissues and mitochondria, with *T.marneffei* found inside the hepatocytes, which were partially degraded. Similar phenomena were observed *invitro*. Therefore, we can infer that *T.marneffei* may bind to unidentified receptors on the surface of hepatocytes that enable active invasion into the cells. Other instances of pathogens that are free-living but can invade cells include: *P.aeruginosa*, which invade epithelial cells (human alveolar epithelial cells and rabbit corneal epithelial cells, etc.) during infection [39]and *Burkholderia pseudomallei*, which invades and survives intracellularly within A549 human lung epithelial cells [40]. In addition, phagocytosis of *A.fumigatus* conidia by epithelial cells has also been demonstrated [41,42]. After 4 h of incubation with *A.fumigatus* showed conidia internalized in amembrane-bound vacuole. The expression levels of factors such as IL-8, TNF-α and GM-CSF were significantly up-regulated [41]. It is worth mentioning that, due to preexisting lung disease, defects in the clearance of internalized pathogens have been shown to increase intracellular survival and further increase the risk of pathogens invading the host and thus predisposing to disease [43–45].

We collected key factors associated with major programmed death modes such as apoptosis, pyroptosis, necroptosis, autophagy and ferroptosis. The results showed that the expression of pyroptosis pathway-related factors was continuously up-regulated and the difference was statistically significant. The activation time of the apoptosis pathway was later and there was no difference in the expression in the early stages. The expression of necroptosis was different, but the change levels were lower than the up-regulated levels of pyroptosis-related factors; also, the expression of key factors of ferroptosis showed the opposite result. On the other hand, we detected factors on different death pathways on AML-12 cells and found that the result of pyroptosis is stable and persistently high expression. Comprehensively, we believe that the acute phase of *T.marneffei* infection is mainly involves the pyroptosis-related pathway. Pyroptosis has emerged as akey mechanism by which inflammasomes promote the host defense against microbial pathogens, but excessive pyroptosis can aggravate the inflammatory response, as observed during septic shock, atherosclerosis, epilepsy and cardiomyopathy, etc [46,47]. With regard to upstream regulators of pyroptosis, avariety of inflammasomes play an important role. In this study, we found that *NLRs* such as *Nlrp1, Nlrp3* and *Nlrc4* were up-regulated by 36.96, 13.98 and 8.97-fold at 7 dpi, respectively, and the expression of *Aim2-like* receptor was up-regulated by 11.51-fold. However, only *Aim2* showed acontinuous upward trend during *invitro* and *invivo* validation (Figure S10). According to the records of KEGG pathway database, AIM2 senses the presence of double-stranded DNA (dsDNA) in the cytoplasm, regardless of whether it is of bacterial, viral or host cellular origin. The role of the AIM2 inflammasome in response to fungi is less clear. Aprevious study showed that AIM2 and NLRP3 cooperatively induced the cytoplasmic inflammasome platform in *A.fumigatus*-infected mouse bone marrow-derived cells. The *Legionella pneumophila* secretory effector protein SDHA is known to inhibit bacterial DNA release and thus interferes with AIM2 inflammasome recognition and activation [48]. Mice lacking both AIM2 and NLRP3 were highly susceptible to aspergillosis, indicating that AIM2 might be co-activated with NLRP3. In this study, we found that fungi could invade liver cells and cause lysis, suggesting that the activation of AIM2 by dsDNA may induce pyroptosis. In addition to AIM2, caspase-4 is another key factor that plays an important role in activating pyroptosis pathway [49]. Independently of the NLRP3 inflammasome and caspase-1, as areceptor for intracellular LPS, caspase-4 promotes pyroptosis through GSDMD cleavage and activation, and through the release of IL-1, IL-18 and HMGB1. However, we showed that *T.marneffei*, athermally dimorphic fungus, could also activate caspase-4. Some studies revealed that mitochondrial permeability transition activated caspase-4 by promoting the assembly of protein complexes (named APAF-1 pyroptosome) to promote pyroptosis [50]. In this study, we found the following using TEM evidence of severe mitochondrial dysfunction and damage in the *T.marneffei* infected group: mitochondrial enlargement, swelling and vacuolar degeneration of the mitochondrial cristae, lighter mitochondrial matrix, and alower overall density of cristae. Therefore, we hypothesized that there might be aconnection between mitochondrial damage and the activation of caspase-4, but the specific mechanism still needs to be further explored.

Numerous factors are responsible for liver injury, such as drugs, alcohol, toxic chemicals, and pathogenic microbes [51]. Many cytokines participate in the development and progression of liver damage, and the interplay between cytokines constitutes acomplex pathogenic process. IL-1β is an important regulator of inflammation, which upregulates the expression of cytokines, including TNF-α and NO, thus amplifying the inflammatory response. IL-17 is acytokine associated with inflammation and autoimmunity and has also been shown to amplify inflammatory responses. However, IL-10 can alleviate inflammatory reactions and inhibit the production and release of many proinflammatory factors. Furthermore, there is evidence that the TGF-β1/STAT3 pathway is an important inflammatory pathway which causes liver fibrosis and cirrhosis [52]. Additionally, various stimuli cause liver damage through activated Tcells and macrophages, which produce ahigh level of inflammatory cytokines, including IL-1β, TNF-α, IFN-γ, and NO, thus ultimately leading to the induction of apoptosis. In this study, we found that in *T.marneffei* infected patients, plasma AST levels were significantly higher than the upper limit of normal (ULN), while ALT exhibited arelatively normal expression level (*P* = 0.531). This distinction may be clinically useful for the diagnosis and treatment of patients infected with *T.marneffei*.

Talaromycosis is asystemic disease with multi-organ involvement. Therefore, anew drug which could inhibit multi-organ injury is urgently required for patients infected with the fungus. VX765, an inhibitor of caspase-1/-4, is an orally absorbed prodrug of the active metabolite and has been demonstrated to be safe for humans as tested in aphase 2b human clinical trial against epilepsy. VX765 reduced disease severity and the expression of inflammatory mediators in models of rheumatoid arthritis and skin inflammation. In this study, we showed that cell damage in *T.marneffei*-infected AML-12 cells was alleviated by VX765, as indicated by decreased expression levels of inflammatory cytokines. Therefore, VX765 may act as apromising agent against multi-organ damage caused by *T.marneffei* infection. Some studies using *caspase-1*^*-/-*^ mice to explore the role of pyroptosis in traumatic injury or noncognate tuberculosis showed that pyroptosis-related proteins were less prominent in *caspase-1*^*-/-*^ mice than those in the WT mice [53,54]. Other studies using intraperitoneal injection of VX765 in an animal pyroptosis model indicated that VX765 at the concentration of 100 or 200 mg/kg could significantly inhibit the expression of IL-1β, IL-18 and GSDMD [55,56]. These *invivo* studies demonstrated that VX765 or *caspase-1*^−/−^ inhibited the expression levels of pyroptosis-related proteins in mice induced by external stimuli (such as *T.marneffei* infection).

However, there are still several limitations that need to be considered further. First, we collected key factors associated with major programmed death modes including apoptosis, pyroptosis, necroptosis, autophagy and ferroptosis. The results showed that the pyroptosis pathway-related factors were continuously up-regulated with adifference that was statistically significant; the activation time of apoptosis pathway was later, and there was no difference in the expression in the early stages; and the expression of necroptosis-related factors was different, but the altered levels were lower than those of pyroptosis-related factors. Therefore, *T.marneffei*-induced cell death might not just be attributed to pyroptosis. In addition, the ES of the apoptosis pathway was relatively low, and the difference was not statistically significant between infection and non-infection groups. Similar to the apoptosis pathway, the necroptosis pathway showed alow ES value, and the difference was not statistically significant between the two groups. GSEA did not find enriched pathways related to ferroptosis and autophagy. According to the results of GSEA, we believed that pyroptosis may be the main cause of cell death after infection with *T.marneffei*, but we cannot draw adefinite conclusion. An additional deficiency of the present study is that our *invitro* data did not fully support the *invivo* data. Overall, the *invitro* and *invivo* data were consistent. At the transcriptional level, the *invitro* results were consistent with the *invivo* data. At protein level, although the *invitro* results were not as significant as those *invivo*, the similar trends were observed. Nonetheless, we acknowledge that this is one of the limitations. Finally, we would like to state that we do not deny the synergistic effect of various cell death modes, especially necroptosis. As shown in Figure5b, NSA appeared to inhibit cell damage more than VX765, which may be another indication that other pathways play arole in *T.marneffei*-mediated liver damage. Cell death is avery complex game in which different core players have the ability to disrupt the delicate balance of the cellular environment from birth to death and from pro-inflammatory to anti-inflammatory signaling. Apoptosis has been the subject of intensive research in the past three decades while pyroptosis has become ahot trend in recent years. Therefore, with the concept of pan-apoptosis (PANoptosis), the research prospect of necroptosis is foreseeable as the last link of the triangle relationship [57,58]. PANoptosis is amultifaceted immune response with important pathophysiological implications for infectious diseases, autoimmunity, and cancer, reflecting the complexity of host responses to pathogens and the interconnectedness of signaling pathways. Subsequent research may be further developed from this perspective.

## Conclusion

In conclusion, *T.marneffei*-infected patients demonstrate abnormal liver function, indicative of liver inflammation and damage. The same phenomenon occurs in the mouse models of *T.marneffei* infection, in which pyroptosis elicited apathological inflammatory response. *T.marneffei* infection induces pyroptosis in hepatocytes through activation of the AIM2-caspase-1/-4-GSDMD axis, which may be an important cause of liver damage. Nevertheless, necroptosis and apoptosis were also activated in infected animal model at14 days post-infection, indicating that death pathways other than pyroptosis may also be involved in the later stage of infection. In addition, we found that *T.marneffei* could invade into the hepatic parenchymal cells, but the entry mechanism needs to be determined in future studies.

## Supplementary Material

Supplemental Material

## Data Availability

The datasets generated for this study are available on request to the corresponding author.
