# Supplementary material for: Talaromyces marneffei activates the AIM2-caspase-1/-4-GSDMD axis to induce pyroptosis in hepatocytes
Source: Virulence. 2022 May 31;13(1):963–79. doi: 10.1080/21505594.2022.2080904 (PMC9176249; doi:10.1080/21505594.2022.2080904)
Supplement: Supplemental Material [file KVIR_A_2080904_SM7735.zip › Supplementary Table 1. Gene-special primers for RT-qPCR.docx]

| **Supplementary Table 1.** Gene-special primers for RT-qPCR | | |
| --- | --- | --- |
| Gene | Forward Primer (5’ to 3’) | Reverse Primer (5’ to 3’) |
| *Aim2* | GTCCTCAAGCTAAGCCTCAGA | CACCGTGACAACAAGTGGAT |
| *caspase-1* | ACAAGGCACGGGACCTATG | TCCCAGTCAGTCCTGGAAATG |
| *caspase-4* | AGCGTTGGGTTTTTGTAGATGC | CCTTGTGAACTCTTCAGGGGA |
| *Il-1**β* | GAAATGCCACCTTTTGACAGTG | TGGATGCTCTCATCAGGACAG |
| *Il-18* | GACTCTTGCGTCAACTTCAAGG | CAGGCTGTCTTTTGTCAACGA |
| *Tnf-α* | CAGGCGGTGCCTATGTCTC | CGATCACCCCGAAGTTCAGTAG |
| *Gsdmd* | TTCCAGTGCCTCCATGAATGT | GCTGTGGACCTCAGTGATCT |
| *Gapdh* | TTCCAGTGCCTCCATGAATGT | GCTGTGGACCTCAGTGATCT |
| *β-actin* | GGCTGTATTCCCCTCCATCG | CCAGTTGGTAACAATGCCATGT |
