# Supplementary material for: Talaromyces marneffei activates the AIM2-caspase-1/-4-GSDMD axis to induce pyroptosis in hepatocytes
Source: Virulence. 2022 May 31;13(1):963–79. doi: 10.1080/21505594.2022.2080904 (PMC9176249; doi:10.1080/21505594.2022.2080904)
Supplement: Supplemental Material [file KVIR_A_2080904_SM7735.zip › Supplemental Table 2.docx]

| **Supplementary Table 2.** Characteristics describe and differential expression analysis of laboratory indexes before and after propensity score adjustment. | | | | | | | | | |
| --- | --- | --- | --- | --- | --- | --- | --- | --- | --- |
| Variables | Before propensity score matched | | | |  | After propensity score matched | | | |
|  | non-Talaromycosis | Talaromycosis | *Z/t* | *P* value |  | non-Talaromycosis | Talaromycosis | *Z/t* | *P* value |
| CD4^+^ count | 190（57，304） | 13（8，49） | -4.842 | **<0.001** |  | 40（15，191） | 13（8，49） | -1.674 | 0.095 |
| neutrophil | 3.28（1.94，5.59） | 2.69（2.22，4.79） | -0.538 | 0.591 |  | 4.35（1.51，8.76） | 2.69（2.22，4.79） | -1.236 | 0.224 |
| leukocyte | 5.21（3.75，7.04） | 3.30（2.72，6.10） | -2.476 | **0.013** |  | 4.92（2.39，7.73） | 3.30（2.72，6.10） | -0.955 | 0.351 |
| erythrocyte | 3.66（3.11，4.23） | 3.13（2.33，3.56） | -2.690 | **0.007** |  | 3.50（2.89，3.98） | 3.13（2.33，3.56） | -1.588 | 0.113 |
| monocyte | 0.49（0.36，0.68） | 0.27（0.15，0.39） | -4.171 | **<0.001** |  | 0.30（0.15，0.49） | 0.27（0.15，0.39） | -0.619 | 0.550 |
| lymphocyte | 1.17（0.66，1.71） | 0.33（0.26，0.64） | -5.272 | **<0.001** |  | 0.38（0.24，0.62） | 0.33（0.26，0.64） | -0.155 | 0.879 |
| thrombocyte | 202（142，273） | 107（58，164） | -3.649 | **<0.001** |  | 140（114，246） | 107（58，164） | -1.799 | 0.074 |
| albumin | 30.03±6.49 | 25.56±3.96 | -8.393 | **<0.001** |  | 30.97±6.70 | 25.56±3.96 | -2.529 | **0.011** |
| AST | 26（20，45） | 59（38，143） | 4.192 | **<0.001** |  | 40（26，60） | 59（38，142） | -2.276 | **0.022** |
| ALT | 20（12，32） | 31（17，58） | 1.970 | **0.049** |  | 29（11，37） | 31（17，58） | -0.632 | 0.531 |
| AST/ALT | 1.47（1.03，2.00） | 2.63（1.49，3.34） | 3.649 | **<0.001** |  | 1.52（1.03，1.85） | 2.63（1.49，3.34） | -2.655 | **0.007** |
| D-dimer | 0.94（0.38，2.11） | 7.19（2.17，12.36） | 5.330 | **<0.001** |  | 1.79（0.67，2.89） | 7.18（2.17，12.36） | -2.938 | **0.003** |
| In normal distribution, the data were presented as mean ± standard deviation (SD), while data with a non-normal distribution were presented as median and interquartile range. | | | | | | | | | |
