# Supplementary figures and images for: Talaromyces marneffei activates the AIM2-caspase-1/-4-GSDMD axis to induce pyroptosis in hepatocytes
Source: Virulence. 2022 May 31;13(1):963–79. doi: 10.1080/21505594.2022.2080904 (PMC9176249; doi:10.1080/21505594.2022.2080904)

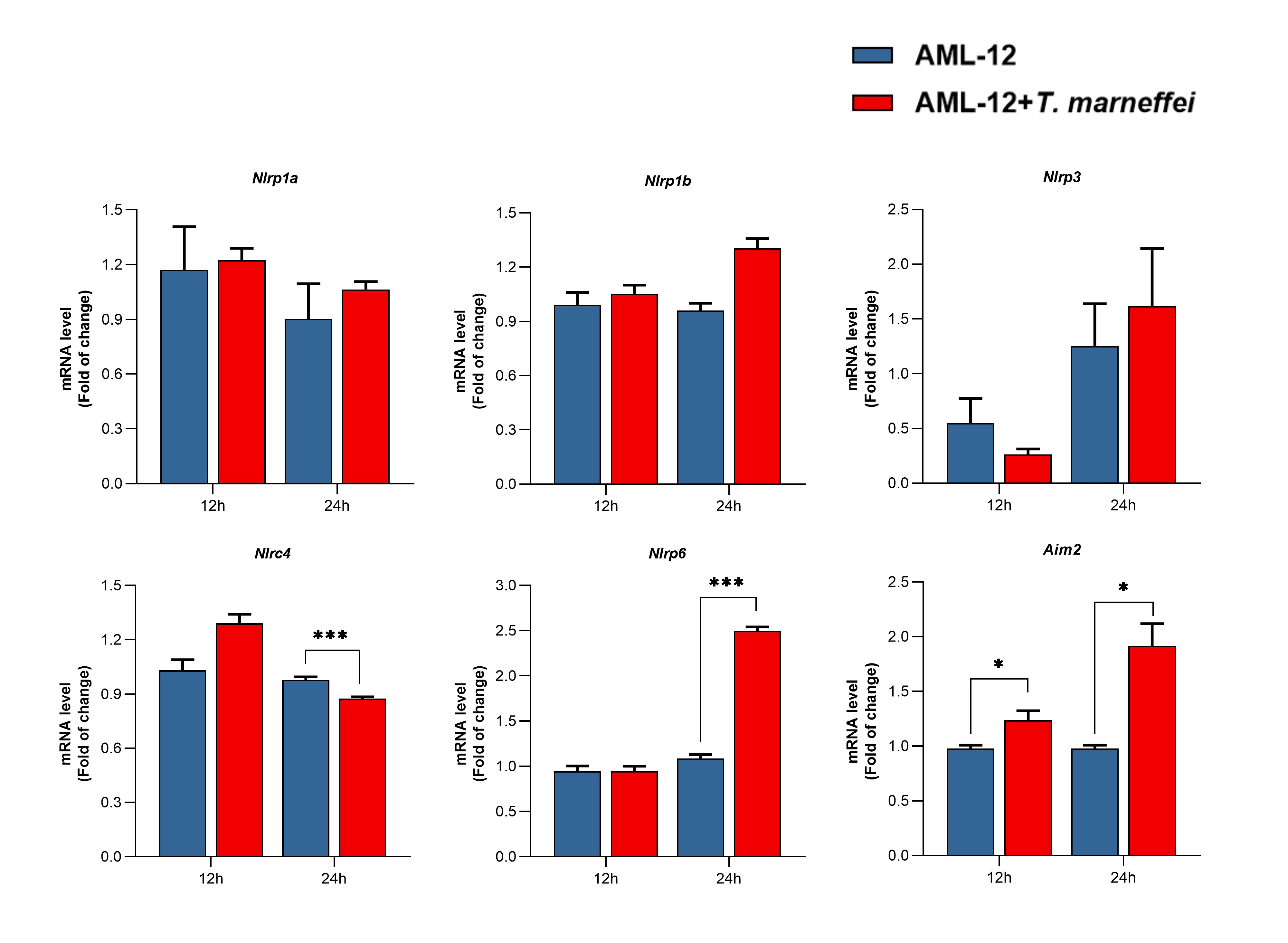

Supplement: Supplemental Material [file KVIR_A_2080904_SM7735.zip › Supplementary Figure 10.JPG]

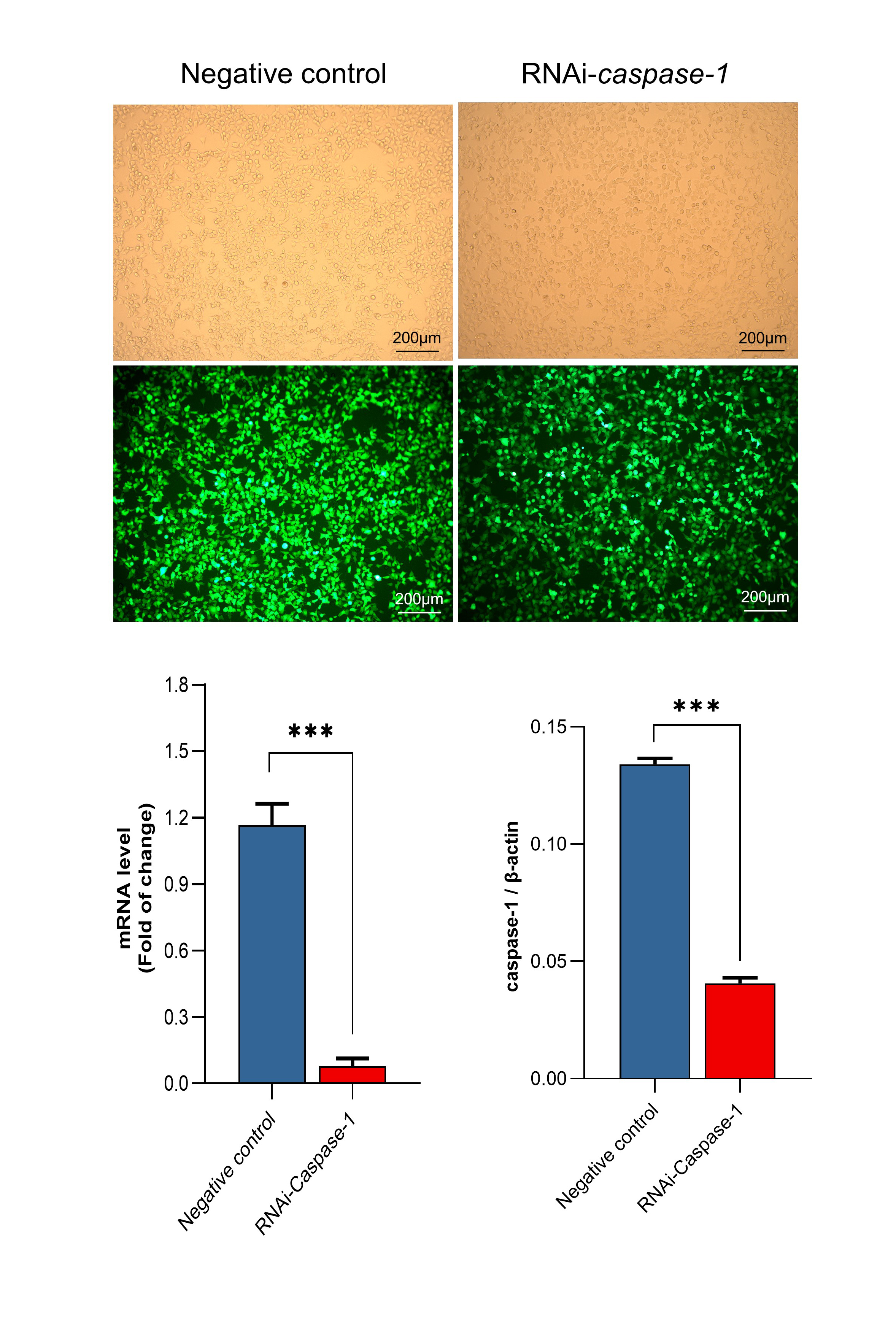

Supplement: Supplemental Material [file KVIR_A_2080904_SM7735.zip › Supplementary Figure 11.JPG]

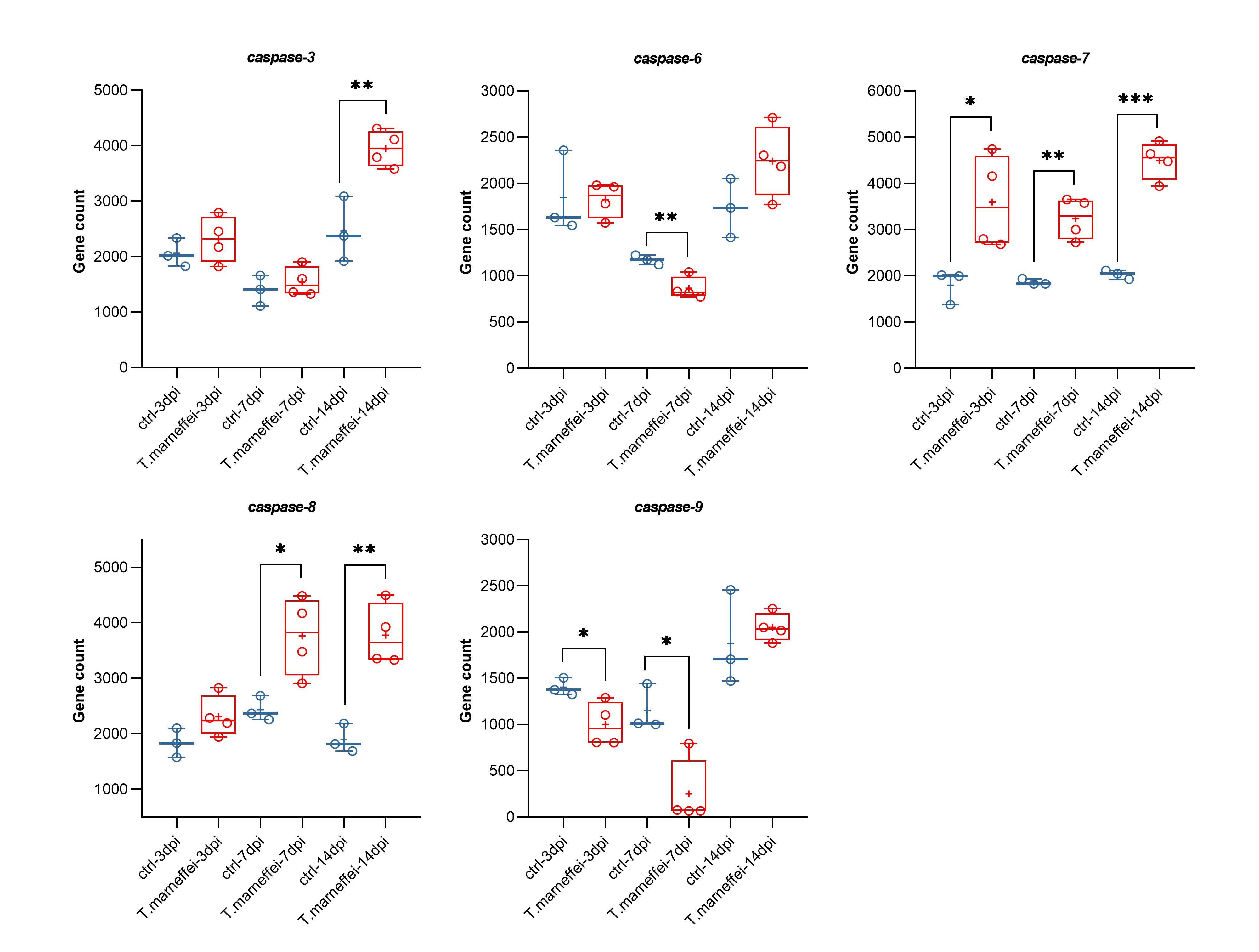

Supplement: Supplemental Material [file KVIR_A_2080904_SM7735.zip › Supplementary Figure 5.JPG]

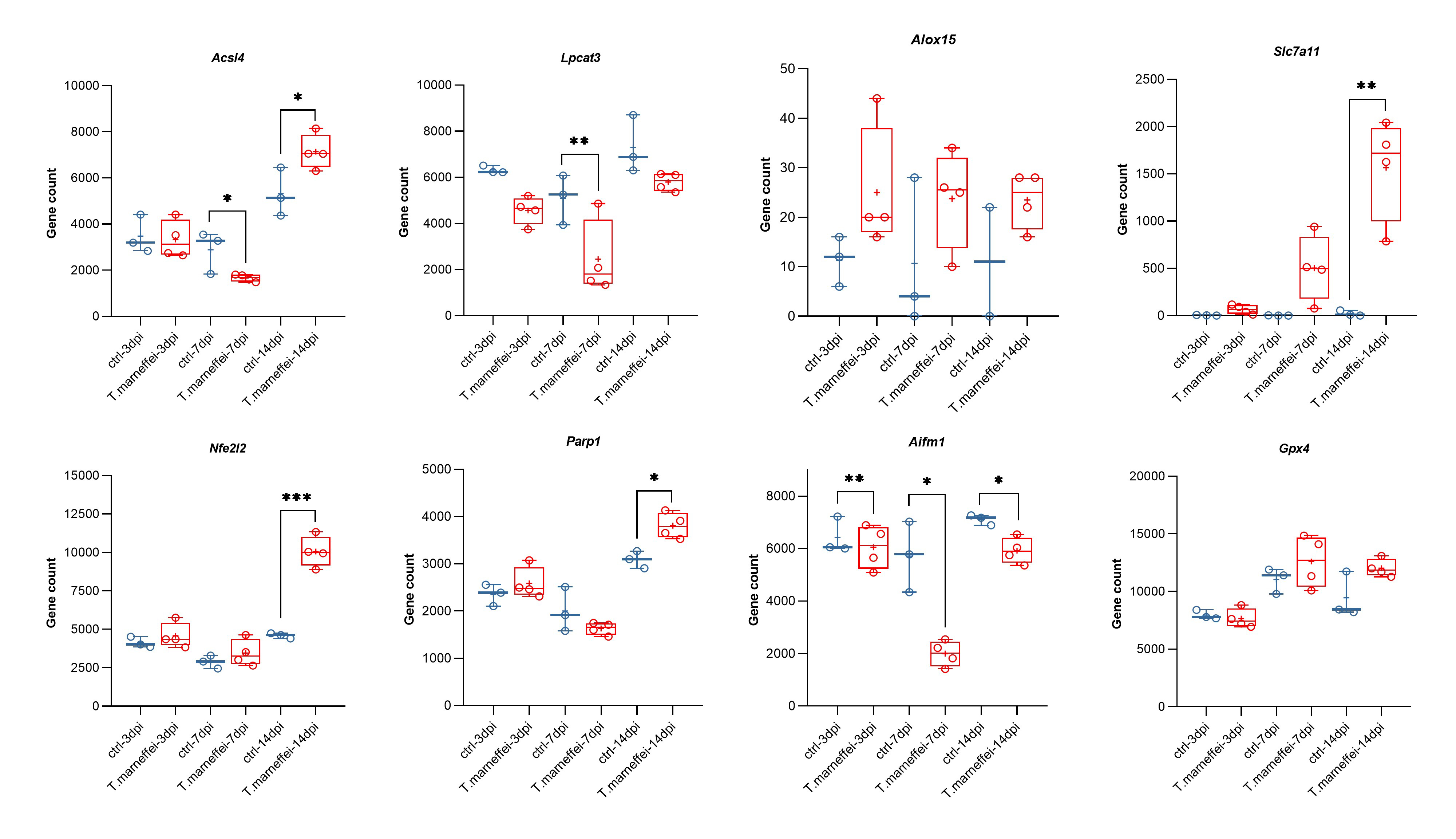

Supplement: Supplemental Material [file KVIR_A_2080904_SM7735.zip › Supplementary Figure 6.JPG]

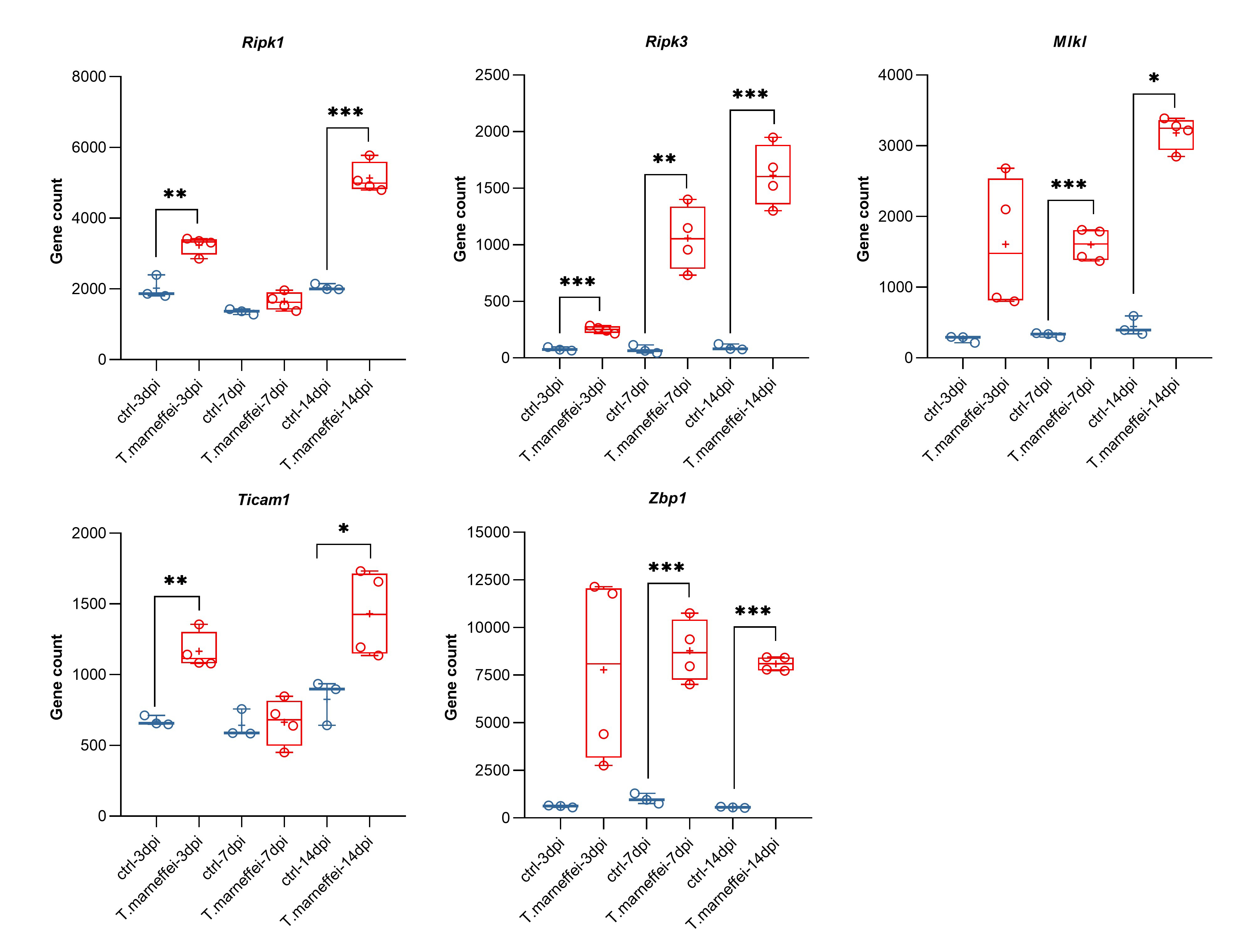

Supplement: Supplemental Material [file KVIR_A_2080904_SM7735.zip › Supplementary Figure 7.JPG]

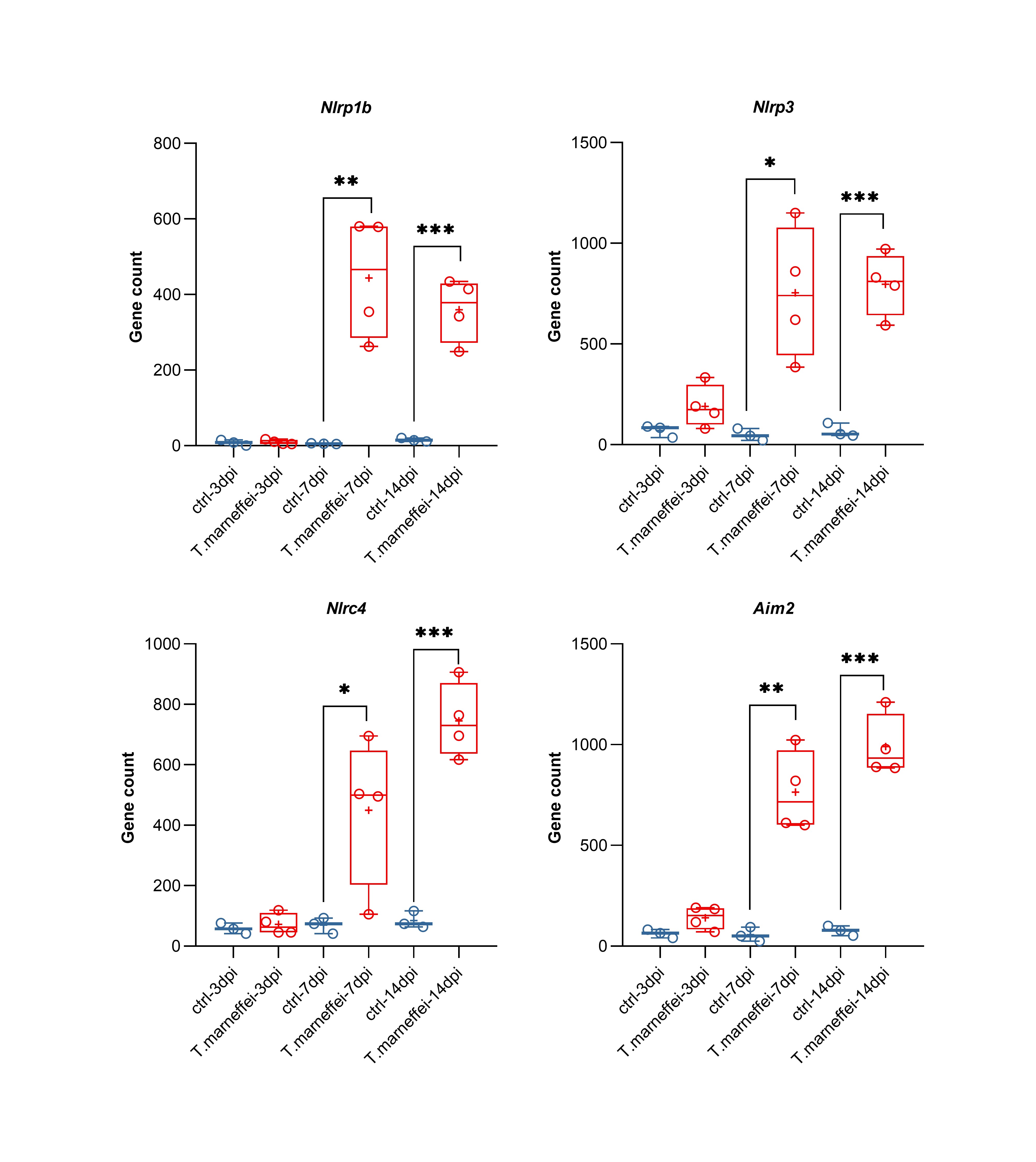

Supplement: Supplemental Material [file KVIR_A_2080904_SM7735.zip › Supplementary Figure 8.JPG]

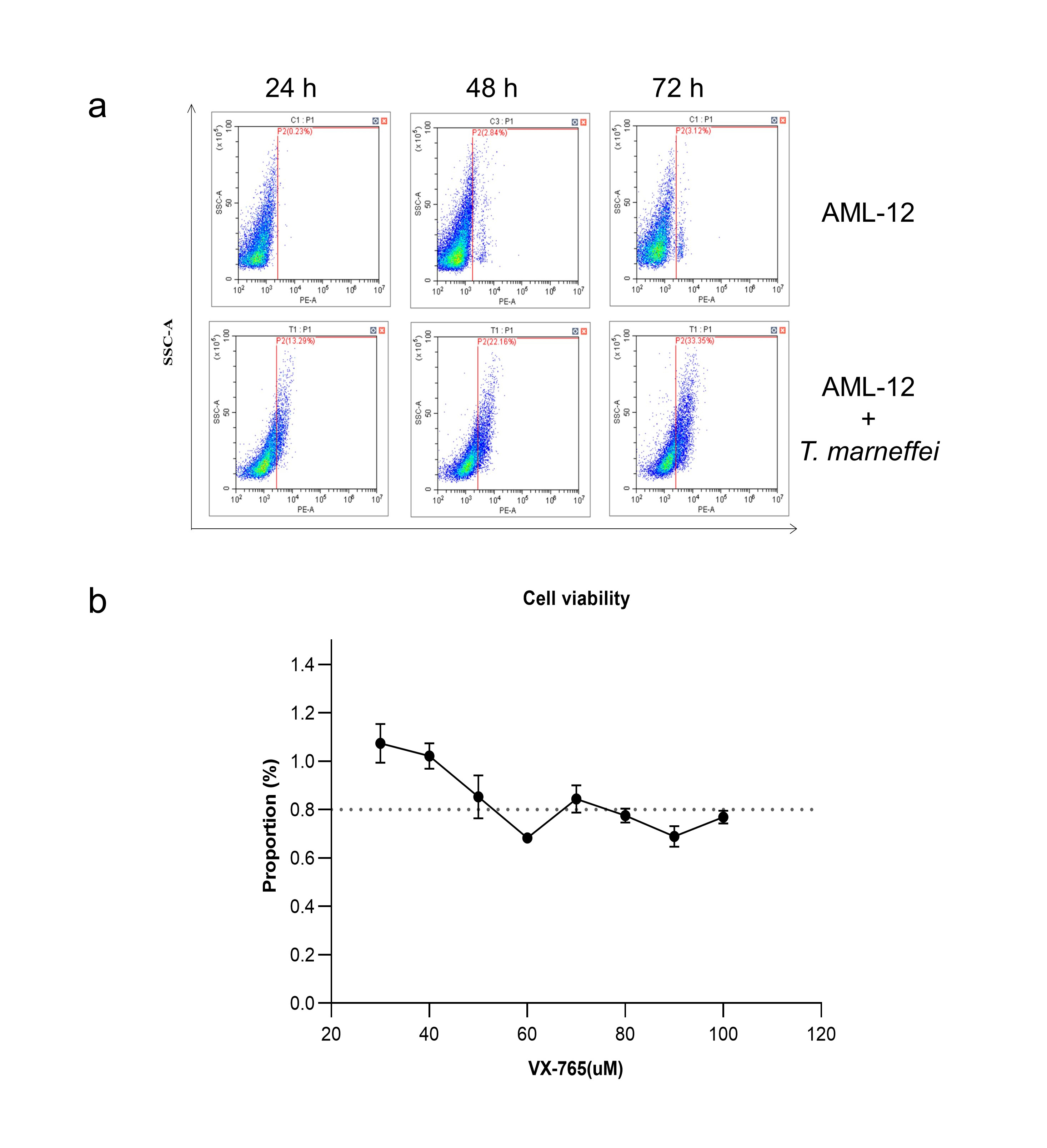

Supplement: Supplemental Material [file KVIR_A_2080904_SM7735.zip › Supplementary Figure 9.JPG]

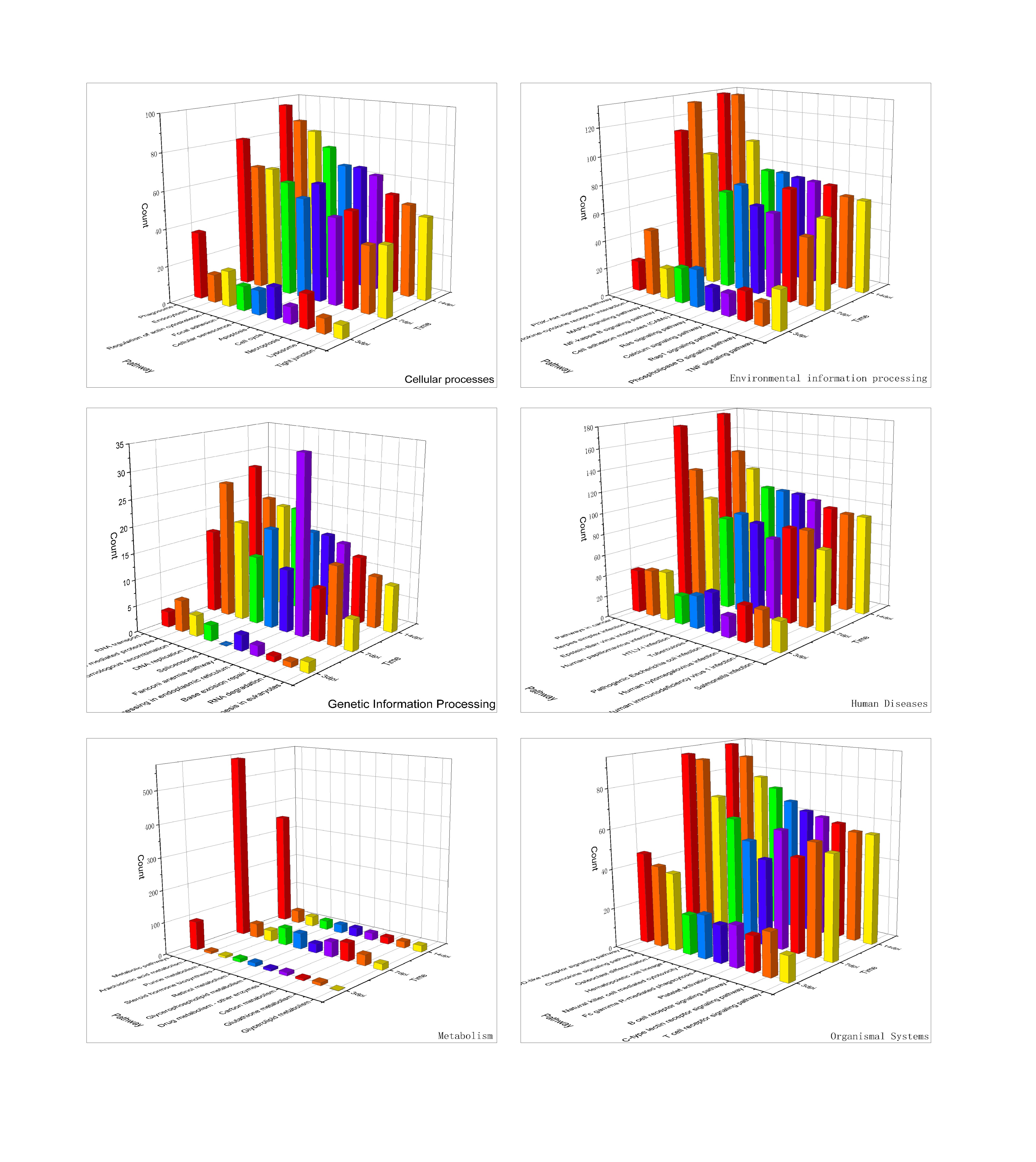

Supplement: Supplemental Material [file KVIR_A_2080904_SM7735.zip › Supplementary Figure 3.JPG]

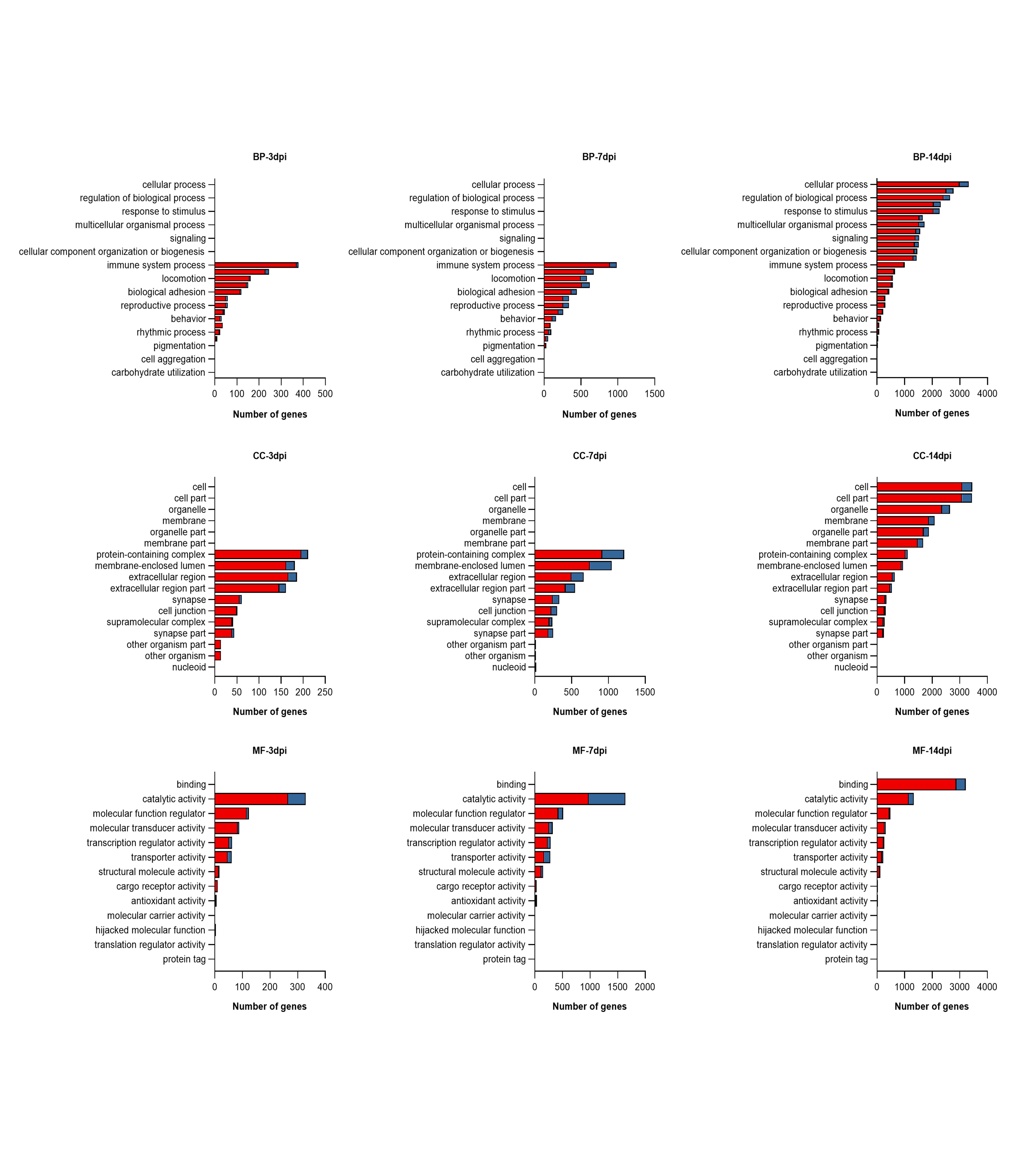

Supplement: Supplemental Material [file KVIR_A_2080904_SM7735.zip › Supplementary_Figure_2.JPG]

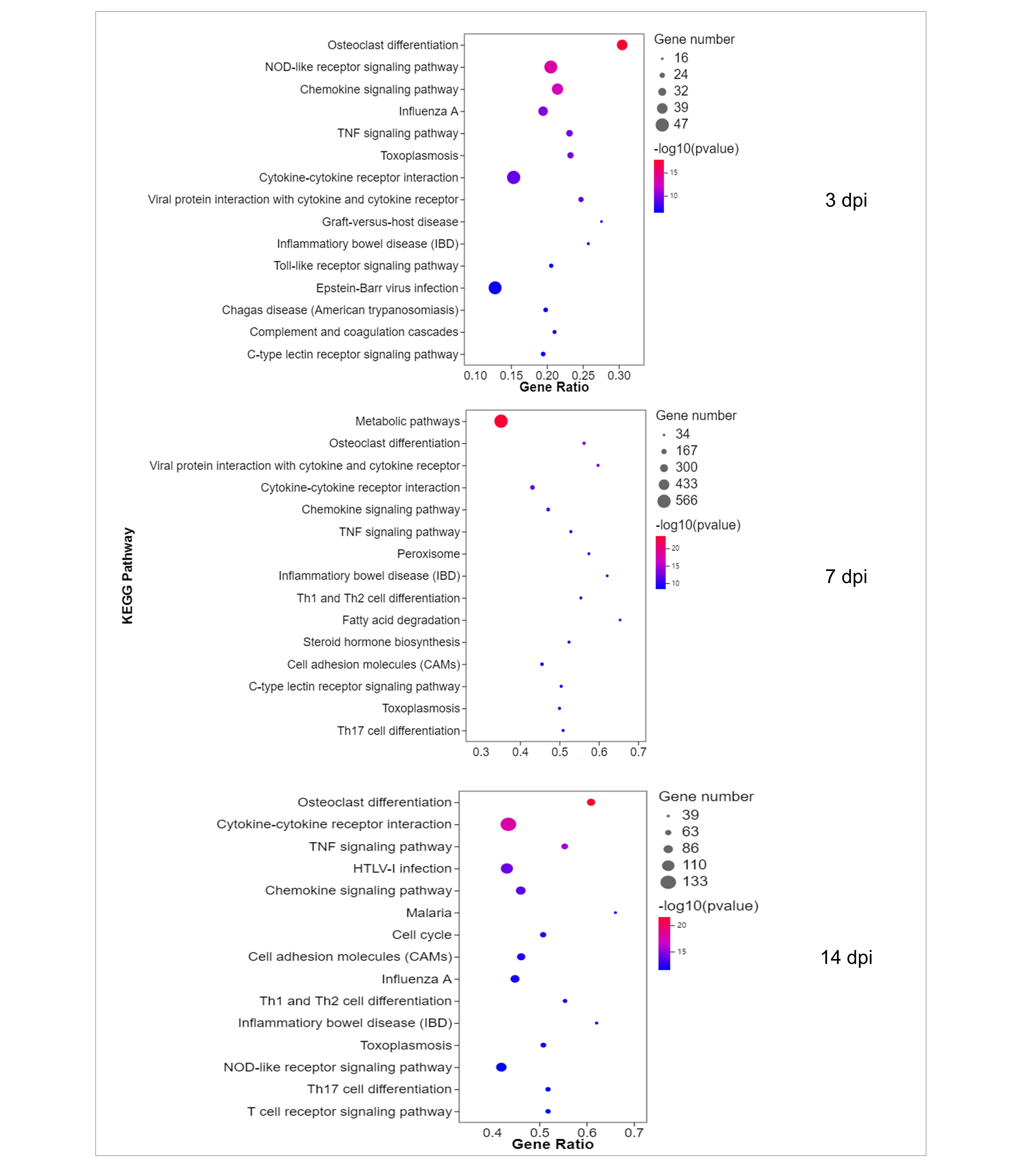

Supplement: Supplemental Material [file KVIR_A_2080904_SM7735.zip › Supplementary Figure 4.JPG]
